# Supplementary material for: A Histone Acetyltransferase Inhibitor with Antifungal Activity against CTG clade Candida Species
Source: Microorganisms. 2019 Jul 15;7(7):201. doi: 10.3390/microorganisms7070201 (PMC6680905; doi:10.3390/microorganisms7070201)
Supplement: Supplementary file 1 [file microorganisms-07-00201-s001.pdf]

Supplementary Information

Table S1: Strains used in this study

Name	Genotype	Reference
C. albicans SC5314	wild-type	[1]
C. albicans CA-MT14	*hat1*Δ/Δ	[2]
C. albicans CA-MT569	*gcn5*Δ/Δ	[3]
C. dubliniensis CD36	wild-type	[4]
C. parapsilosis ATCC22019	wild-type	www.attc.org
C. krusei ATCC6258	wild-type	www.attc.org
C. tropicalis AKH2143	wild-type	clinical isolate
C. guillermondii AKH2119	wild-type	clinical isolate
C. kefyr AKH2057	wild-type	clinical isolate
C. lipolytica AKH2269	wild-type	clinical isolate
C. lusitaniae AKH2124	wild-type	clinical isolate
C. glabrata ATCC2001	wild-type	www.attc.org
S. cerevisiae BY4741	MATa *his3*Δ1 *leu2*Δ0 *LYS2* *met15*Δ0 *ura3*Δ0	[5]

1. Gillum, A.M.; Tsay, E.Y.H.; Kirsch, D.R. Isolation of the Candida albicans gene for orotidine-5'-phosphate decarboxylase by complementation of S. cerevisiae ura3 and E. coli pyrF mutations. *Molec Gen Genet* **1984**, *198*, 179–182.
2. Tscherner, M.; Stappler, E.; Hnisz, D.; Kuchler, K. The histone acetyltransferase Hat1 facilitates DNA damage repair and morphogenesis in Candida albicans. *Molecular Microbiology* **2012**, *86*, 1197–1214.
3. Shivarathri, R.; Tscherner, M.; Zwolanek, F.; Singh, N.K.; Chauhan, N.; Kuchler, K. The Fungal Histone Acetyl Transferase Gcn5 Controls Virulence of the Human Pathogen Candida albicans through Multiple Pathways. *Scientific Reports* **2019**, *9, 9445*.
4. Sullivan, D.J.; Westerneng, T.J.; Haynes, K.A.; Bennett, D.E.; Coleman, D.C. Candida dubliniensis sp. nov.: phenotypic and molecular characterization of a novel species associated with oral candidosis in HIV-infected individuals. *Microbiology* **1995**, *141*, 1507–1521.
5. Brachmann, C.B.; Davies, A.; Cost, G.J.; Caputo, E.; Li, J.; Hieter, P.; Boeke, J.D. Designer deletion strains derived from Saccharomyces cerevisiae S288C: A useful set of strains and plasmids for PCR-mediated gene disruption and other applications. *Yeast* **1998**, *14*, 115–132.
